# μ-Pyrazine-2,5-dicarboxyl­ato-bis­[chlorido(η^6^-*p*-cymene)ruthenium(II)] *tert*-butanol disolvate

**DOI:** 10.1107/S1600536808000202

**Published:** 2008-01-09

**Authors:** Noelia M. Sanchez Ballester, Mark R. J. Elsegood, Martin B. Smith

**Affiliations:** a Chemistry Department, Loughborough University, Loughborough, Leicestershire LE11 3TU, England

## Abstract

A new *tert*-butanol solvate of [{(^*i*^PrC_6_H_4_Me)RuCl}_2_{μ-2,5-pyz(COO)_2_}] (pyz = pyrazine) has been crystallized and structurally characterized. The solvate, [Ru_2_(C_10_H_14_)_2_(C_6_H_2_N_2_O_4_)Cl_2_]·2C_4_H_10_O, contains one half-mol­ecule of the ruthenium(II) complex and one mol­ecule of *tert*-butanol in the asymmetric unit. The complex mol­ecule lies on an inversion centre with the two chlorides *trans*. In contrast, the previously reported structure was solvent-free. Similar metric parameters are found between the butanol solvate and the solvent-free form and an inter­molecular O—H⋯O hydrogen bond exists between μ-pyrazine-2,5-dicarboxyl­ato-bis­[chlorido(η^6^-*p*-cymene)­ruthenium(II)] and the *tert*-butanol mol­ecule.

## Related literature

The structure of the solvent-free complex has been reported previously (Govindaswamy *et al.*, 2007 ▶). One mol­ecule adopts a *trans* configuration of the two chloro ligands while the second lies on a twofold axis giving the two chloro ligands a *cis *configuration. For other related literature, see: Cadierno *et al.* (2002 ▶); Carter *et al.* (1993 ▶); Dann *et al.* (2006 ▶); Dorcier *et al.* (2005 ▶); Drommi *et al.* (1995 ▶); Ganter (2003 ▶); Gemel *et al.* (2000 ▶); Grote *et al.* (2004 ▶); Ion *et al.* (2006 ▶); Konar *et al.* (2004 ▶); Lahuerta *et al.* (1988 ▶); Ma *et al.* (2004 ▶); Pinto *et al.* (2004 ▶).
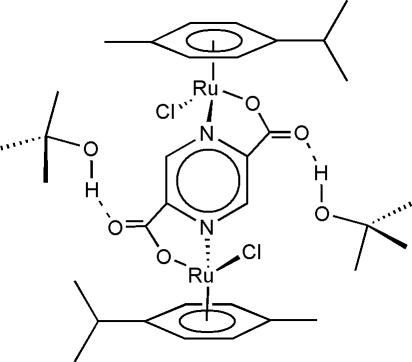


## Experimental

### 

#### Crystal data

[Ru_2_(C_10_H_14_)_2_(C_6_H_2_N_2_O_4_)Cl_2_]·2C_4_H_10_O
*M* *_r_* = 855.80
Monoclinic, 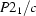

*a* = 9.8483 (2) Å
*b* = 11.3968 (3) Å
*c* = 16.3448 (3) Å
β = 91.465 (2)°
*V* = 1833.93 (7) Å^3^
*Z* = 2
Mo *K*α radiation
μ = 1.01 mm^−1^
*T* = 120 (2) K
0.18 × 0.04 × 0.03 mm

#### Data collection

Bruker–Nonius Kappa APEXII diffractometer
Absorption correction: multi-scan (*SADABS*; Sheldrick, 2007 ▶) *T*_min_ = 0.839, *T*_max_ = 0.970
18492 measured reflections
4195 independent reflections
3594 reflections with *I* > 2σ(*I*)
*R*_int_ = 0.046

#### Refinement

*R*[*F* ^2^ > 2σ(*F* ^2^)] = 0.049
*wR*(*F* ^2^) = 0.115
*S* = 1.06
4196 reflections
215 parameters
H-atom parameters constrained
Δρ_max_ = 2.78 e Å^−3^
Δρ_min_ = −1.04 e Å^−3^

### 

Data collection: *COLLECT* (Hooft, 1998 ▶); cell refinement: *DENZO* (Otwinowski & Minor, 1997 ▶) and *COLLECT*; data reduction: *DENZO* and *COLLECT*; program(s) used to solve structure: *SHELXTL* (Sheldrick, 2001 ▶); program(s) used to refine structure: *SHELXTL*; molecular graphics: *SHELXTL*; software used to prepare material for publication: *SHELXTL* and local programs.

## Supplementary Material

Crystal structure: contains datablocks global, I. DOI: 10.1107/S1600536808000202/bv2088sup1.cif

Structure factors: contains datablocks I. DOI: 10.1107/S1600536808000202/bv2088Isup2.hkl

Additional supplementary materials:  crystallographic information; 3D view; checkCIF report

## Figures and Tables

**Table 1:** Hydrogen-bond geometry (Å, °)

*D*—H⋯*A*	*D*—H	H⋯*A*	*D*⋯*A*	*D*—H⋯*A*
O3—H3⋯O1	0.84	1.98	2.804 (5)	168

**Fig. 1.**
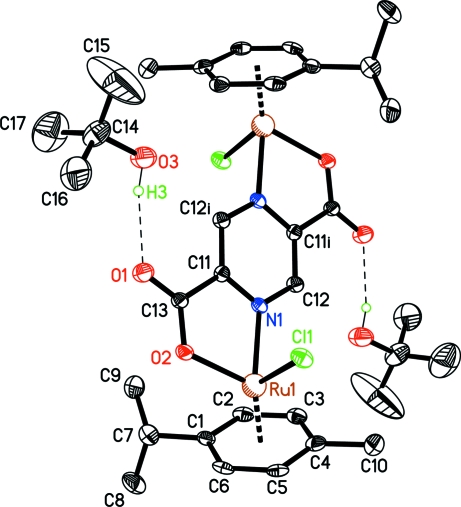
View of 1, showing the atom-labelling scheme. Displacement ellipsoids are drawn at the 50% probability level. All H atoms except on O(3) have been removed for clarity. Symmetry operator: A = -x, -y, -z.

## supplementary crystallographic information

### Comment

There has been considerable interest in the chemistry of areneruthenium(II) complexes for a variety of purposes. These range from their interesting and varied coordination chemistry (Cadierno *et al.*, 2002; Drommi *et al.*, 1995) including DNA binding studies (Dorcier *et al.*, 2005) to applications in areas including supramolecular chemistry, as highly selective receptors and catalysis (Dann *et al.*, 2006; Ganter, 2003; Grote *et al.*, 2004; Ion *et al.*, 2006). These organometallic ruthenium(II) fragments have also been used in the synthesis of chiral half-sandwich compounds (Ganter, 2003; Pinto *et al.*, 2004). Pyrazine polycarboxylic acids are excellent ligands for metal coordination (Konar *et al.*, 2004; Ma *et al.*, 2004). Complexes of ruthenium(II) with pyrazine carboxylic acids are known and their redox behaviour has been studied by voltammetric methods (Govindaswamy *et al.*, 2007). We report here the molecular structure of a new *tert*-butanol solvate of the ruthenium(II) complex [{(η^6^-*p*-*^i^*PrC_6_H_4_Me)RuCl}_2_{µ-2,5-pyz(COO)_2_}]\. *^t^*BuOH 1. The solvent free structure, 2, which contains one molecule with a *trans* configuration of the two chloro ligands and a second molecule with twofold symmetry that has two chloro ligands disposed in a *cis* configuration, has recently been reported (Govindaswamy *et al.*, 2007).

The molecular structure of 1 is shown in Figure 1 and shows a typical piano-stool geometry at each ruthenium(II) centre with each metal bonded to an η^6^-*p*-*^i^*PrC_6_H_4_Me arene [Ru—C_centroid_ 1.6689 (16) Å], a terminal chloride and a dianionic *N*,*O*-chelating pyrazine ligand. The Ru—Cl bond length in 1 [2.3975 (11) Å] is slightly shorter than that in the *trans* isomer [2.408 (5) Å] of 2 yet similar to the *cis* isomer [2.388 (3) Å, 2.399 (3) Å]. The Ru—O and Ru—N bond distances in 1 [2.099 (3) Å and 2.097 (3) Å respectively] are similar to those in the *cis* isomer of 2 [2.083 (10)/2.109 (9) Å and 2.102 (7)/2.074 (7) Å respectively] and with those of other related three-legged piano-stool ruthenium(II) complexes (Carter *et al.*, 1993; Gemel *et al.*, 2000; Lahuerta *et al.*, 1988). The N(1)—Ru(1)—O(2) bite angle in 1 [77.29 (12)°] is broadly as expected for this type of five-membered chelating ligand. The η^6^-*p*-*^i^*PrC_6_H_4_Me arene ring is essentially planar with C—C bond lengths in the range 1.392 (6)–1.435 (6) Å. The Ru complex is hydrogen-bonded to a *^t^*BuOH molecule through a strong intermolecular O—H···O interaction.

In summary, we have reported the crystal structure of a new *tert*-butanol solvate form of [{(η^6^-*p*-*^i^*PrC_6_H_4_Me)RuCl}_2_{µ-2,5-pyz(COO)_2_}] 1 that displays very similar bond lengths and angles around the ruthenium(II) coordination sphere to complex 2 recently published (Govindaswamy *et al.*, 2007).

### Experimental

Crystals of compound 1 were obtained unexpectedly from the experimental procedure outlined here. Boronic acid (0.004 g, 0.007 mmol) in warm *^t^*BuOH (10 ml) was added dropwise to a solution of [{(η^6^-*p*-*^i^*PrC_6_H_4_Me)RuCl}_2_{µ-2,5-pyz(COO)_2_}] (0.023 g, 0.0325 mmol) in CH_2_Cl_2_ (10 ml) affording an orange-red solution. The solution was stirred at room temperature for 3 h and the volume was concentrated to 2–3 ml. Suitable X-ray quality crystals of 1 were obtained by slow vapour diffusion of diethyl ether into the concentrated CH_2_Cl_2_/*^t^*BuOH solution.

### Refinement

H atoms were placed in geometric positions (C—H distance = 0.95 Å for aryl H; 0.98 Å for methine, 1.00 Å for methyl H; and 0.84 Å for O—H) using a riding model. *U*_iso_ values were set to 1.2*U*_eq _(C) (1.5*U*_eq _(C/O)for methyl H and OH atoms respectively).

### Crystal data

**Table:**

[Ru_2_(C_10_H_14_)_2_(C_6_H_2_N_2_O_4_)Cl_2_]·2(C_4_H_10_O)	*F*_000_ = 876
*M**_r_* = 855.80	*D*_x_ = 1.550 Mg m^−^^3^
Monoclinic, *P*2_1_/*c*	Mo *K*α radiation λ = 0.71073 Å
Hall symbol: -P 2ybc	Cell parameters from 4336 reflections
*a* = 9.8483 (2) Å	θ = 2.9–27.5º
*b* = 11.3968 (3) Å	µ = 1.01 mm^−^^1^
*c* = 16.3448 (3) Å	*T* = 120 (2) K
β = 91.465 (2)º	Rod, brown
*V* = 1833.93 (7) Å^3^	0.18 × 0.04 × 0.03 mm
*Z* = 2	

### Data collection

**Table:**

Bruker Nonius APEXII CCD camera on κ-goniostat diffractometer	4195 independent reflections
Radiation source: Bruker-Nonius FR591 rotating anode	3594 reflections with *I* > 2σ(*I*)
Monochromator: graphite	*R*_int_ = 0.046
Detector resolution: 4090x4096pixels/62x62mm pixels mm^-1^	θ_max_ = 27.5º
*T* = 120(2) K	θ_min_ = 3.0º
φ and ω scans	*h* = −12→12
Absorption correction: multi-scan(SADABS; Sheldrick, 2007)	*k* = −14→14
*T*_min_ = 0.839, *T*_max_ = 0.970	*l* = −20→21
18492 measured reflections	

### Refinement

**Table:**

Refinement on *F*^2^	Secondary atom site location: difference Fourier map
Least-squares matrix: full	Hydrogen site location: inferred from neighbouring sites
*R*[*F*^2^ > 2σ(*F*^2^)] = 0.049	H-atom parameters constrained
*wR*(*F*^2^) = 0.115	*w* = 1/[σ^2^(*F*_o_^2^) + (0.0329*P*)^2^ + 10.5824*P*] where *P* = (*F*_o_^2^ + 2*F*_c_^2^)/3
*S* = 1.06	(Δ/σ)_max_ = 0.034
4196 reflections	Δρ_max_ = 2.78 e Å^−^^3^
215 parameters	Δρ_min_ = −1.04 e Å^−^^3^
Primary atom site location: structure-invariant direct methods	Extinction correction: none

### Special details

**Table:**

Geometry. All e.s.d.'s (except the e.s.d. in the dihedral angle between two l.s. planes) are estimated using the full covariance matrix. The cell e.s.d.'s are taken into account individually in the estimation of e.s.d.'s in distances, angles and torsion angles; correlations between e.s.d.'s in cell parameters are only used when they are defined by crystal symmetry. An approximate (isotropic) treatment of cell e.s.d.'s is used for estimating e.s.d.'s involving l.s. planes.
Refinement. Refinement of *F*^2^ against ALL reflections. The weighted *R*-factor *wR* and goodness of fit *S* are based on *F*^2^, conventional *R*-factors *R* are based on *F*, with *F* set to zero for negative *F*^2^. The threshold expression of *F*^2^ > σ(*F*^2^) is used only for calculating *R*-factors(gt) *etc*. and is not relevant to the choice of reflections for refinement. *R*-factors based on *F*^2^ are statistically about twice as large as those based on *F*, and *R*- factors based on ALL data will be even larger.

### Fractional atomic coordinates and isotropic or equivalent isotropic displacement parameters (Å^2^)

**Table:**

	*x*	*y*	*z*	*U*_iso_*/*U*_eq_	
Ru1	0.09960 (3)	0.28799 (3)	0.037062 (19)	0.01586 (11)	
Cl1	0.13823 (11)	0.29072 (9)	−0.10715 (6)	0.0245 (2)	
C1	0.0607 (4)	0.3713 (4)	0.1547 (2)	0.0199 (8)	
C2	0.1442 (4)	0.2730 (4)	0.1682 (2)	0.0206 (8)	
H2	0.1190	0.2153	0.2069	0.025*	
C3	0.2668 (4)	0.2590 (4)	0.1243 (3)	0.0231 (9)	
H3A	0.3213	0.1912	0.1335	0.028*	
C4	0.3085 (4)	0.3447 (4)	0.0673 (3)	0.0236 (9)	
C5	0.2248 (4)	0.4460 (4)	0.0561 (3)	0.0217 (8)	
H5	0.2516	0.5056	0.0193	0.026*	
C6	0.1045 (4)	0.4591 (4)	0.0982 (3)	0.0216 (8)	
H6	0.0506	0.5272	0.0893	0.026*	
C7	−0.0741 (5)	0.3876 (4)	0.1953 (3)	0.0281 (10)	
H7	−0.1365	0.4297	0.1560	0.034*	
C8	−0.0512 (6)	0.4661 (5)	0.2700 (3)	0.0360 (11)	
H8A	0.0074	0.4256	0.3103	0.054*	
H8B	−0.1387	0.4839	0.2944	0.054*	
H8C	−0.0076	0.5393	0.2533	0.054*	
C9	−0.1430 (5)	0.2730 (5)	0.2199 (3)	0.0382 (12)	
H9A	−0.1494	0.2205	0.1725	0.057*	
H9B	−0.2344	0.2899	0.2392	0.057*	
H9C	−0.0894	0.2352	0.2638	0.057*	
C10	0.4356 (5)	0.3291 (5)	0.0202 (3)	0.0363 (12)	
H10A	0.5109	0.3694	0.0487	0.054*	
H10B	0.4225	0.3623	−0.0347	0.054*	
H10C	0.4565	0.2453	0.0159	0.054*	
N1	0.0476 (3)	0.1115 (3)	0.01791 (19)	0.0155 (6)	
C11	−0.0800 (4)	0.0943 (3)	−0.0104 (2)	0.0161 (7)	
C12	0.1276 (4)	0.0178 (3)	0.0281 (2)	0.0177 (8)	
H12	0.2185	0.0280	0.0478	0.021*	
C13	−0.1656 (4)	0.2038 (3)	−0.0213 (2)	0.0171 (8)	
O1	−0.2818 (3)	0.1959 (3)	−0.0502 (2)	0.0293 (7)	
O2	−0.1073 (3)	0.2977 (2)	0.00366 (18)	0.0205 (6)	
C14	−0.5253 (5)	0.0313 (5)	−0.1783 (3)	0.0391 (12)	
C16	−0.5410 (7)	0.1587 (6)	−0.2005 (5)	0.0596 (18)	
H16A	−0.4918	0.1749	−0.2506	0.089*	
H16B	−0.5041	0.2073	−0.1558	0.089*	
H16C	−0.6375	0.1768	−0.2095	0.089*	
C17	−0.6231 (8)	0.0080 (8)	−0.1086 (5)	0.081 (2)	
H17A	−0.7153	0.0310	−0.1260	0.122*	
H17B	−0.5948	0.0538	−0.0604	0.122*	
H17C	−0.6218	−0.0757	−0.0949	0.122*	
C15	−0.5355 (14)	−0.0404 (12)	−0.2492 (7)	0.176 (8)	
H15A	−0.4682	−0.0151	−0.2887	0.264*	
H15B	−0.6268	−0.0333	−0.2739	0.264*	
H15C	−0.5184	−0.1224	−0.2340	0.264*	
O3	−0.3929 (4)	0.0100 (3)	−0.1422 (3)	0.0438 (10)	
H3	−0.3711	0.0663	−0.1114	0.066*	

### Atomic displacement parameters (Å^2^)

**Table:**

	*U*^11^	*U*^22^	*U*^33^	*U*^12^	*U*^13^	*U*^23^
Ru1	0.01907 (17)	0.01018 (16)	0.01823 (17)	0.00040 (12)	−0.00136 (11)	−0.00046 (12)
Cl1	0.0329 (5)	0.0211 (5)	0.0194 (5)	0.0014 (4)	0.0014 (4)	−0.0001 (4)
C1	0.0221 (19)	0.0154 (19)	0.022 (2)	−0.0005 (15)	−0.0016 (16)	−0.0046 (16)
C2	0.028 (2)	0.017 (2)	0.0160 (19)	−0.0009 (16)	−0.0094 (16)	−0.0006 (15)
C3	0.023 (2)	0.019 (2)	0.027 (2)	0.0026 (16)	−0.0100 (17)	−0.0034 (17)
C4	0.023 (2)	0.023 (2)	0.025 (2)	−0.0061 (17)	−0.0033 (16)	−0.0053 (17)
C5	0.029 (2)	0.0116 (18)	0.025 (2)	−0.0073 (16)	−0.0016 (17)	−0.0005 (16)
C6	0.031 (2)	0.0112 (19)	0.022 (2)	−0.0005 (16)	−0.0028 (17)	−0.0057 (16)
C7	0.030 (2)	0.028 (2)	0.027 (2)	0.0027 (19)	0.0020 (18)	−0.0011 (19)
C8	0.049 (3)	0.031 (3)	0.029 (2)	0.003 (2)	0.010 (2)	−0.006 (2)
C9	0.032 (3)	0.039 (3)	0.044 (3)	−0.004 (2)	0.012 (2)	−0.005 (2)
C10	0.025 (2)	0.040 (3)	0.044 (3)	−0.005 (2)	0.004 (2)	−0.012 (2)
N1	0.0189 (15)	0.0120 (15)	0.0155 (15)	0.0026 (12)	0.0002 (12)	0.0012 (12)
C11	0.0156 (17)	0.0163 (18)	0.0163 (18)	0.0013 (14)	−0.0009 (14)	0.0015 (15)
C12	0.0193 (18)	0.0127 (18)	0.021 (2)	0.0029 (14)	−0.0025 (15)	0.0024 (15)
C13	0.0175 (18)	0.0118 (18)	0.022 (2)	0.0012 (14)	−0.0015 (15)	0.0016 (15)
O1	0.0230 (15)	0.0192 (16)	0.045 (2)	0.0034 (12)	−0.0097 (14)	−0.0013 (14)
O2	0.0194 (14)	0.0138 (14)	0.0280 (16)	0.0032 (11)	−0.0038 (11)	−0.0024 (12)
C14	0.033 (3)	0.045 (3)	0.039 (3)	−0.004 (2)	−0.014 (2)	0.010 (2)
C16	0.048 (4)	0.052 (4)	0.077 (5)	0.013 (3)	−0.018 (3)	0.006 (3)
C17	0.073 (5)	0.079 (6)	0.092 (6)	−0.014 (4)	0.014 (5)	0.027 (5)
C15	0.225 (14)	0.180 (13)	0.117 (9)	0.140 (12)	−0.123 (10)	−0.100 (9)
O3	0.0337 (19)	0.0284 (19)	0.068 (3)	0.0077 (15)	−0.0194 (18)	−0.0112 (18)

### Geometric parameters (Å, °)

**Table:**

Ru1—N1	2.097 (3)	C9—H9B	0.9800
Ru1—O2	2.099 (3)	C9—H9C	0.9800
Ru1—C3	2.176 (4)	C10—H10A	0.9800
Ru1—C2	2.184 (4)	C10—H10B	0.9800
Ru1—C1	2.187 (4)	C10—H10C	0.9800
Ru1—C6	2.191 (4)	N1—C12	1.335 (5)
Ru1—C5	2.200 (4)	N1—C11	1.343 (5)
Ru1—C4	2.201 (4)	C11—C12^i^	1.389 (5)
Ru1—Cl1	2.3975 (11)	C11—C13	1.514 (5)
C1—C2	1.404 (6)	C12—C11^i^	1.389 (5)
C1—C6	1.435 (6)	C12—H12	0.9500
C1—C7	1.511 (6)	C13—O1	1.231 (5)
C2—C3	1.429 (6)	C13—O2	1.277 (5)
C2—H2	0.9500	C14—C15	1.419 (10)
C3—C4	1.417 (6)	C14—O3	1.438 (6)
C3—H3A	0.9500	C14—C16	1.503 (8)
C4—C5	1.428 (6)	C14—C17	1.534 (9)
C4—C10	1.496 (7)	C16—H16A	0.9800
C5—C6	1.392 (6)	C16—H16B	0.9800
C5—H5	0.9500	C16—H16C	0.9800
C6—H6	0.9500	C17—H17A	0.9800
C7—C8	1.525 (6)	C17—H17B	0.9800
C7—C9	1.530 (7)	C17—H17C	0.9800
C7—H7	1.0000	C15—H15A	0.9800
C8—H8A	0.9800	C15—H15B	0.9800
C8—H8B	0.9800	C15—H15C	0.9800
C8—H8C	0.9800	O3—H3	0.8400
C9—H9A	0.9800		
			
N1—Ru1—O2	77.29 (12)	C5—C6—Ru1	71.9 (2)
N1—Ru1—C3	97.48 (14)	C1—C6—Ru1	70.7 (2)
O2—Ru1—C3	153.12 (15)	C5—C6—H6	119.5
N1—Ru1—C2	96.53 (14)	C1—C6—H6	119.5
O2—Ru1—C2	115.40 (14)	Ru1—C6—H6	130.7
C3—Ru1—C2	38.27 (17)	C1—C7—C8	108.1 (4)
N1—Ru1—C1	120.00 (14)	C1—C7—C9	114.3 (4)
O2—Ru1—C1	90.86 (13)	C8—C7—C9	110.4 (4)
C3—Ru1—C1	68.67 (16)	C1—C7—H7	108.0
C2—Ru1—C1	37.47 (15)	C8—C7—H7	108.0
N1—Ru1—C6	157.55 (15)	C9—C7—H7	108.0
O2—Ru1—C6	94.69 (14)	C7—C8—H8A	109.5
C3—Ru1—C6	80.13 (16)	C7—C8—H8B	109.5
C2—Ru1—C6	67.69 (15)	H8A—C8—H8B	109.5
C1—Ru1—C6	38.28 (15)	C7—C8—H8C	109.5
N1—Ru1—C5	160.06 (15)	H8A—C8—H8C	109.5
O2—Ru1—C5	122.04 (14)	H8B—C8—H8C	109.5
C3—Ru1—C5	67.62 (16)	C7—C9—H9A	109.5
C2—Ru1—C5	80.04 (16)	C7—C9—H9B	109.5
C1—Ru1—C5	68.30 (16)	H9A—C9—H9B	109.5
C6—Ru1—C5	36.97 (16)	C7—C9—H9C	109.5
N1—Ru1—C4	122.58 (15)	H9A—C9—H9C	109.5
O2—Ru1—C4	159.86 (14)	H9B—C9—H9C	109.5
C3—Ru1—C4	37.78 (17)	C4—C10—H10A	109.5
C2—Ru1—C4	68.83 (16)	C4—C10—H10B	109.5
C1—Ru1—C4	81.87 (16)	H10A—C10—H10B	109.5
C6—Ru1—C4	68.07 (16)	C4—C10—H10C	109.5
C5—Ru1—C4	37.87 (16)	H10A—C10—H10C	109.5
N1—Ru1—Cl1	84.84 (9)	H10B—C10—H10C	109.5
O2—Ru1—Cl1	85.47 (9)	C12—N1—C11	118.1 (3)
C3—Ru1—Cl1	120.66 (13)	C12—N1—Ru1	127.4 (3)
C2—Ru1—Cl1	158.93 (12)	C11—N1—Ru1	114.5 (3)
C1—Ru1—Cl1	153.45 (11)	N1—C11—C12^i^	121.0 (4)
C6—Ru1—Cl1	115.75 (12)	N1—C11—C13	115.7 (3)
C5—Ru1—Cl1	91.50 (12)	C12^i^—C11—C13	123.3 (3)
C4—Ru1—Cl1	92.66 (12)	N1—C12—C11^i^	121.0 (4)
C2—C1—C6	118.2 (4)	N1—C12—H12	119.5
C2—C1—C7	123.1 (4)	C11^i^—C12—H12	119.5
C6—C1—C7	118.6 (4)	O1—C13—O2	126.2 (4)
C2—C1—Ru1	71.2 (2)	O1—C13—C11	119.6 (3)
C6—C1—Ru1	71.0 (2)	O2—C13—C11	114.2 (3)
C7—C1—Ru1	128.0 (3)	C13—O2—Ru1	117.7 (2)
C1—C2—C3	120.6 (4)	C15—C14—O3	106.4 (6)
C1—C2—Ru1	71.4 (2)	C15—C14—C16	110.8 (7)
C3—C2—Ru1	70.6 (2)	O3—C14—C16	110.4 (5)
C1—C2—H2	119.7	C15—C14—C17	118.3 (9)
C3—C2—H2	119.7	O3—C14—C17	104.3 (5)
Ru1—C2—H2	131.2	C16—C14—C17	106.4 (6)
C4—C3—C2	121.1 (4)	C14—C16—H16A	109.5
C4—C3—Ru1	72.1 (2)	C14—C16—H16B	109.5
C2—C3—Ru1	71.2 (2)	H16A—C16—H16B	109.5
C4—C3—H3A	119.5	C14—C16—H16C	109.5
C2—C3—H3A	119.5	H16A—C16—H16C	109.5
Ru1—C3—H3A	129.9	H16B—C16—H16C	109.5
C3—C4—C5	117.7 (4)	C14—C17—H17A	109.5
C3—C4—C10	121.1 (4)	C14—C17—H17B	109.5
C5—C4—C10	121.2 (4)	H17A—C17—H17B	109.5
C3—C4—Ru1	70.2 (2)	C14—C17—H17C	109.5
C5—C4—Ru1	71.0 (2)	H17A—C17—H17C	109.5
C10—C4—Ru1	129.7 (3)	H17B—C17—H17C	109.5
C6—C5—C4	121.3 (4)	C14—C15—H15A	109.5
C6—C5—Ru1	71.2 (2)	C14—C15—H15B	109.5
C4—C5—Ru1	71.1 (2)	H15A—C15—H15B	109.5
C6—C5—H5	119.4	C14—C15—H15C	109.5
C4—C5—H5	119.4	H15A—C15—H15C	109.5
Ru1—C5—H5	131.3	H15B—C15—H15C	109.5
C5—C6—C1	121.1 (4)	C14—O3—H3	109.5
			
N1—Ru1—C1—C2	57.5 (3)	Cl1—Ru1—C4—C10	−26.2 (4)
O2—Ru1—C1—C2	133.3 (2)	C3—C4—C5—C6	−1.5 (6)
C3—Ru1—C1—C2	−28.8 (2)	C10—C4—C5—C6	178.2 (4)
C6—Ru1—C1—C2	−130.2 (4)	Ru1—C4—C5—C6	52.6 (4)
C5—Ru1—C1—C2	−102.3 (3)	C3—C4—C5—Ru1	−54.1 (3)
C4—Ru1—C1—C2	−65.6 (3)	C10—C4—C5—Ru1	125.5 (4)
Cl1—Ru1—C1—C2	−145.2 (2)	N1—Ru1—C5—C6	−147.6 (4)
N1—Ru1—C1—C6	−172.3 (2)	O2—Ru1—C5—C6	47.9 (3)
O2—Ru1—C1—C6	−96.5 (2)	C3—Ru1—C5—C6	−103.8 (3)
C3—Ru1—C1—C6	101.4 (3)	C2—Ru1—C5—C6	−66.0 (3)
C2—Ru1—C1—C6	130.2 (4)	C1—Ru1—C5—C6	−28.8 (2)
C5—Ru1—C1—C6	27.9 (2)	C4—Ru1—C5—C6	−134.2 (4)
C4—Ru1—C1—C6	64.7 (3)	Cl1—Ru1—C5—C6	133.5 (2)
Cl1—Ru1—C1—C6	−14.9 (4)	N1—Ru1—C5—C4	−13.4 (6)
N1—Ru1—C1—C7	−60.3 (4)	O2—Ru1—C5—C4	−178.0 (2)
O2—Ru1—C1—C7	15.5 (4)	C3—Ru1—C5—C4	30.3 (3)
C3—Ru1—C1—C7	−146.6 (4)	C2—Ru1—C5—C4	68.2 (3)
C2—Ru1—C1—C7	−117.8 (5)	C1—Ru1—C5—C4	105.3 (3)
C6—Ru1—C1—C7	112.0 (5)	C6—Ru1—C5—C4	134.2 (4)
C5—Ru1—C1—C7	139.9 (4)	Cl1—Ru1—C5—C4	−92.4 (2)
C4—Ru1—C1—C7	176.6 (4)	C4—C5—C6—C1	0.3 (6)
Cl1—Ru1—C1—C7	97.1 (4)	Ru1—C5—C6—C1	52.9 (3)
C6—C1—C2—C3	−2.4 (6)	C4—C5—C6—Ru1	−52.6 (3)
C7—C1—C2—C3	176.3 (4)	C2—C1—C6—C5	1.7 (6)
Ru1—C1—C2—C3	52.6 (3)	C7—C1—C6—C5	−177.0 (4)
C6—C1—C2—Ru1	−55.0 (3)	Ru1—C1—C6—C5	−53.4 (3)
C7—C1—C2—Ru1	123.6 (4)	C2—C1—C6—Ru1	55.1 (3)
N1—Ru1—C2—C1	−132.7 (2)	C7—C1—C6—Ru1	−123.6 (4)
O2—Ru1—C2—C1	−53.7 (3)	N1—Ru1—C6—C5	151.4 (3)
C3—Ru1—C2—C1	133.5 (4)	O2—Ru1—C6—C5	−140.9 (2)
C6—Ru1—C2—C1	30.7 (2)	C3—Ru1—C6—C5	65.7 (3)
C5—Ru1—C2—C1	67.2 (3)	C2—Ru1—C6—C5	103.5 (3)
C4—Ru1—C2—C1	104.8 (3)	C1—Ru1—C6—C5	133.7 (4)
Cl1—Ru1—C2—C1	134.7 (3)	C4—Ru1—C6—C5	28.3 (2)
N1—Ru1—C2—C3	93.8 (2)	Cl1—Ru1—C6—C5	−53.7 (3)
O2—Ru1—C2—C3	172.8 (2)	N1—Ru1—C6—C1	17.7 (5)
C1—Ru1—C2—C3	−133.5 (4)	O2—Ru1—C6—C1	85.4 (2)
C6—Ru1—C2—C3	−102.8 (3)	C3—Ru1—C6—C1	−68.0 (2)
C5—Ru1—C2—C3	−66.3 (3)	C2—Ru1—C6—C1	−30.1 (2)
C4—Ru1—C2—C3	−28.7 (2)	C5—Ru1—C6—C1	−133.7 (4)
Cl1—Ru1—C2—C3	1.2 (5)	C4—Ru1—C6—C1	−105.3 (3)
C1—C2—C3—C4	1.2 (6)	Cl1—Ru1—C6—C1	172.66 (19)
Ru1—C2—C3—C4	54.2 (3)	C2—C1—C7—C8	97.1 (5)
C1—C2—C3—Ru1	−53.0 (3)	C6—C1—C7—C8	−84.3 (5)
N1—Ru1—C3—C4	135.8 (3)	Ru1—C1—C7—C8	−171.7 (3)
O2—Ru1—C3—C4	−147.6 (3)	C2—C1—C7—C9	−26.2 (6)
C2—Ru1—C3—C4	−133.1 (4)	C6—C1—C7—C9	152.5 (4)
C1—Ru1—C3—C4	−104.8 (3)	Ru1—C1—C7—C9	65.0 (5)
C6—Ru1—C3—C4	−66.8 (3)	O2—Ru1—N1—C12	−177.7 (4)
C5—Ru1—C3—C4	−30.4 (3)	C3—Ru1—N1—C12	−24.5 (4)
Cl1—Ru1—C3—C4	47.4 (3)	C2—Ru1—N1—C12	−63.0 (3)
N1—Ru1—C3—C2	−91.1 (2)	C1—Ru1—N1—C12	−94.1 (4)
O2—Ru1—C3—C2	−14.5 (4)	C6—Ru1—N1—C12	−106.7 (5)
C1—Ru1—C3—C2	28.3 (2)	C5—Ru1—N1—C12	15.7 (6)
C6—Ru1—C3—C2	66.3 (3)	C4—Ru1—N1—C12	6.0 (4)
C5—Ru1—C3—C2	102.7 (3)	Cl1—Ru1—N1—C12	95.8 (3)
C4—Ru1—C3—C2	133.1 (4)	O2—Ru1—N1—C11	4.7 (3)
Cl1—Ru1—C3—C2	−179.5 (2)	C3—Ru1—N1—C11	157.9 (3)
C2—C3—C4—C5	0.8 (6)	C2—Ru1—N1—C11	119.3 (3)
Ru1—C3—C4—C5	54.6 (3)	C1—Ru1—N1—C11	88.2 (3)
C2—C3—C4—C10	−178.9 (4)	C6—Ru1—N1—C11	75.6 (5)
Ru1—C3—C4—C10	−125.1 (4)	C5—Ru1—N1—C11	−161.9 (4)
C2—C3—C4—Ru1	−53.8 (3)	C4—Ru1—N1—C11	−171.7 (3)
N1—Ru1—C4—C3	−55.1 (3)	Cl1—Ru1—N1—C11	−81.8 (3)
O2—Ru1—C4—C3	135.3 (4)	C12—N1—C11—C12^i^	0.3 (6)
C2—Ru1—C4—C3	29.0 (3)	Ru1—N1—C11—C12^i^	178.2 (3)
C1—Ru1—C4—C3	65.4 (3)	C12—N1—C11—C13	−179.6 (3)
C6—Ru1—C4—C3	102.6 (3)	Ru1—N1—C11—C13	−1.8 (4)
C5—Ru1—C4—C3	130.3 (4)	C11—N1—C12—C11^i^	−0.3 (6)
Cl1—Ru1—C4—C3	−140.7 (2)	Ru1—N1—C12—C11^i^	−177.9 (3)
N1—Ru1—C4—C5	174.6 (2)	N1—C11—C13—O1	176.9 (4)
O2—Ru1—C4—C5	5.0 (6)	C12^i^—C11—C13—O1	−3.0 (6)
C3—Ru1—C4—C5	−130.3 (4)	N1—C11—C13—O2	−4.7 (5)
C2—Ru1—C4—C5	−101.3 (3)	C12^i^—C11—C13—O2	175.3 (4)
C1—Ru1—C4—C5	−64.9 (3)	O1—C13—O2—Ru1	−172.8 (4)
C6—Ru1—C4—C5	−27.7 (2)	C11—C13—O2—Ru1	9.0 (4)
Cl1—Ru1—C4—C5	89.0 (2)	N1—Ru1—O2—C13	−7.8 (3)
N1—Ru1—C4—C10	59.4 (5)	C3—Ru1—O2—C13	−89.2 (4)
O2—Ru1—C4—C10	−110.3 (5)	C2—Ru1—O2—C13	−99.1 (3)
C3—Ru1—C4—C10	114.5 (5)	C1—Ru1—O2—C13	−128.4 (3)
C2—Ru1—C4—C10	143.5 (5)	C6—Ru1—O2—C13	−166.6 (3)
C1—Ru1—C4—C10	179.9 (5)	C5—Ru1—O2—C13	166.9 (3)
C6—Ru1—C4—C10	−143.0 (5)	C4—Ru1—O2—C13	163.3 (4)
C5—Ru1—C4—C10	−115.2 (5)	Cl1—Ru1—O2—C13	77.9 (3)

Symmetry codes: (i) −*x*, −*y*, −*z*.

### Hydrogen-bond geometry (Å, °)

**Table:**

*D*—H···*A*	*D*—H	H···*A*	*D*···*A*	*D*—H···*A*
O3—H3···O1	0.84	1.98	2.804 (5)	168
